# GSDMD-mediated pyroptosis: a critical mechanism of diabetic nephropathy

**DOI:** 10.1017/erm.2021.27

**Published:** 2021-12-27

**Authors:** Yi Zuo, Li Chen, Huiping Gu, Xiaoyun He, Zhen Ye, Zhao Wang, Qixiang Shao, Caiping Xue

**Affiliations:** 1 Department of Geriatrics, Affiliated Huai'an No.2 People's Hospital of Xuzhou Medical University, No.62, Huaihai South Road, Qingjiangpu District, Huai'an 223300, China; 2 Guangxi Key Laboratory of Brain and Cognitive Neuroscience, Guilin Medical University, Guilin 541001, China; 3 Department of Electrophysiology, Affiliated Huai'an No.1 People's Hospital of Nanjing Medical University, No.1 the Yellow River West Road, Huaiyin District, Huai'an 223300, China; 4 Departments of Ultrasound Imaging, Xiangya Hospital, Central South University, Changsha, Hunan 410008, China; 5 Jiangsu College of Nursing, No.2 the Yellow River West Road, Huai'an 223300, China; 6 Department of Immunology, School of Medicine, Jiangsu University, Zhenjiang 212013, China

**Keywords:** Diabetic nephropathy, GSDMD, podocytes, pyroptosis, tubular epithelial cells

## Abstract

Pyroptosis is a recently identified mechanism of programmed cell death related to Caspase-1 that triggers a series of inflammatory reactions by releasing several proinflammatory factors such as IL-1β and IL-18. The process is characterised by the rupture of cell membranes and the release of cell contents through the mediation of gasdermin (GSDM) proteins. GSDMD is an important member of the GSDM family and plays a critical role in the two pathways of pyroptosis. Diabetic nephropathy (DN) is a microvascular complication of diabetes and a major cause of end-stage renal disease. Recently, it was revealed that GSDMD-mediated pyroptosis plays an important role in the occurrence and development of DN. In this review, we focus on two types of kidney cells, tubular epithelial cells and renal podocytes, to illustrate the mechanism of pyroptosis in DN and provide new ideas for the prevention, early diagnosis and molecular therapy of DN.

## Introduction

Diabetic nephropathy (DN), one of the main complications of diabetes mellitus (DM), is also the main cause of end-stage renal disease (ESRD) (Ref. 1).

So far, it has been found that cell death exists in the process of DN, such as apoptosis, necrosis, autophagy, pyroptosis, ferroptosis and so on (Refs 2–6). Apoptosis and necrosis were first discovered to be involved in the exploration of the mechanism of DN, whose occurrence is closely related to tumour necrosis factor-*α* (TNF-*α*) (Refs 7–13). TNF is a cytokine from the proinflammatory cytokine family (Refs 10, 11). TNF-*α*, IL-1 and IL-6 are all inflammatory factors released during cell death (Ref. 14). TNF-*α*, the transmembrane protein, is not only expressed by immune cells, such as monocytes/macrophages (including microglia in the nervous system), B cells, activated T and NK cells, but also by a diverse array of non-immune cells, such as fibroblasts, endothelial cells, epithelial cells and tumour cells (Ref. 15). Transmembrane TNF-*α* assembles into homotrimers, which are cleaved by matrix metalloproteinase TNF-*α* converting enzyme (TACE/ADAM17), leading to the releasing of TNF-*α* homotrimers, which is responsible for the endocrine function of TNF-*α* (Refs 16, 17). Both forms bind to structurally related but functionally distinct receptors: TNF receptor 1 (TNFR1), which is ubiquitously expressed in almost any cell type at a low level (Refs 18, 19). From the mechanism perspective, TNFR1 is involved in mediating extrinsic apoptosis (Ref. 20). After binding to the ligand TNF, the TNFR1 signalling complex separates from the plasma membrane. In the cytoplasm, the FAS-associated death domain protein (FADD)-bound initiator caspase-8 is recruited to the complex and interacts with receptor-interacting protein kinase 1 (RIPK1) (Refs 21, 22). After caspase-8 is activated, RIPK1 activity is blocked by proteolytic cleavage of RIPK1 by caspase-8 (Ref. 23). Caspase-8 with proteolytic activity is involved in the activation of apoptosis and pyroptosis pathways (Ref. 9).

Pyroptosis, which is a newly discovered type of programmed cell death, has received increasing attention in recent years. Upon the recognition of ‘danger signals’ when the body is exposed to infection, the innate immune system initiates a response that may trigger cell necrosis, apoptosis or pyroptosis to kill the invading microorganisms. As a lytic and inflammatory death that depends on inflammasomes and caspases, pyroptosis is characterised by the rupture of the plasma membrane, swelling and dissolution of cells, which is mediated by the gasdermin (GSDM) protein family. Finally, it leads to the delivery of proinflammatory factors such as IL-1*β* and IL-18, and the release of cell contents. GSDMD plays a critical role in pyroptosis; therefore, pyroptosis is also defined as GSDM-mediated programmed necrotic cell death (Ref. 24).

Relevant studies have reported that pyroptosis is involved in the occurrence and development of DN (Refs 25–27). To a certain extent, inhibiting the occurrence of pyroptosis equals to alleviating the damage to the kidney in DN. Currently, the pyroptosis signalling pathways involved in the pathogenesis of DN constitute a hot topic. In this review, we summarise the mechanisms of pyroptosis in two types of intrinsic renal cells, tubular epithelial cells (TECs) and renal podocytes, during DN.

## The role of GSDMD in pyroptosis

### GSDM family

The GSDM family, a protein family with sequence homology, mainly includes GSDM A, B, C, D, E, DFNB59 (Refs 28–30). Most of them can form oligomer and insert into the cellular or mitochondrial membranes to form a hole, except DFNB59, which does not have a domain within a pore-forming activity (Ref. 31).

Both GSDMA and GSDMB are located at human chromosome 17q21.1, while GSDMC and GSDMD at chromosome 8q24. Mice lack Gsdmb, but carry genes encoding three homologs of GSDMA (Gsdma1–3) clustered on chromosome 11, and four homologs of GASDMC (Gsdmc1–4) clustered on chromosome 15 (Ref. 32).

**Fig. 1.**
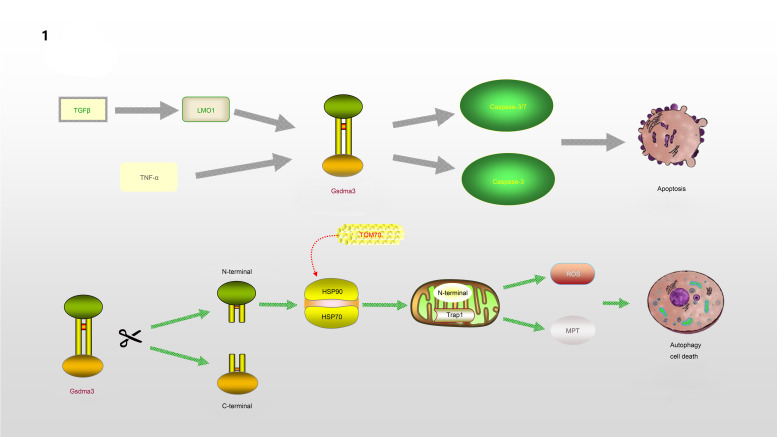
GSDMA is involved in the process of regulating apoptosis and autophagy: TNF-*α* or TGF-*β*, LMO1 target to Gsdma3, accompanied by the release of Caspase3/7, leading to the occurrence of apoptosis. The cleavage of Gsdma3 promotes the release of N-terminal and C-terminal. The deletion or mutation of C-terminal makes N-terminal have the ability to participate in the regulation of autophagy. N-terminal interacts with HSP90 and translocates to mitochondria through Tom70, and then associates with Trap1, which results in the massive release of ROS and mitochondrial permeability transition.

Gsdma3/GSDMA participates in the regulation of cellular apoptosis, autophagy and oxidative stress (OS) (Ref. 32). Lei *et al* (Ref. 33). found that TNF-*α* directly leads to the release of Gsdma3, enhancing caspase-3 expression and causing apoptosis. GSDMA, a downstream protein in transforming growth factor-*β* (TGF-*β*)-dependent apoptosis, is a potential suppressor of gastric cancer, indicating that GSDMA suppression is required for tumorigenesis in gastric tissue (Ref. 34). LMO1 (LIM domain only 1), a member of the LMO protein family, is a transcriptional regulator. It has different expression patterns in embryonic and adult tissues, indicating that it plays a vital role in expressing different cellular biological functions (Ref. 35). Without the ability of DNA binding, it is believed to interact with other molecules through its LIM domain when playing a role in transcriptional regulation. LMO1 has been confirmed to regulate the expression of GSDM family proteins (Ref. 36). LMO1 targets GSDMA through Runt-related transcription factor 3 and participates in the apoptosis induced by TGF-*β*, which is accompanied by the release of caspase-3/7 (Ref. 36). In addition, autophagy can be induced by mutations in the Gsdma3 C-terminal domain (Ref. 37). The loss or mutation of the C-terminal domain results in the release of the intrinsic pro-autophagic capability of the N-terminal domain. The N-terminal domain associates with Hsp90 and migrates to mitochondria through the mitochondrial importer receptor Tom70, where it interacts with the mitochondrial chaperone Trap1 and causes an increase in the production of reactive oxygen species (ROS) and in mitochondrial permeability transition (Ref. 38). The specific regulation process of GSDMA is shown in Figure 1.

**Fig. 2.**
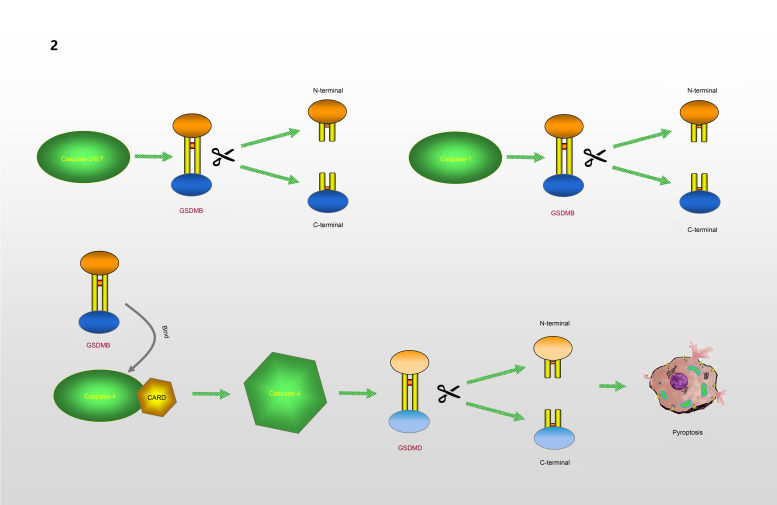
GSDMB causes pyroptosis: GSDMB is a substrate of Caspase-3/6/7 and could be cleaved into N-terminal and C-terminal. GSDMB could be cleaved by Caspase-1 at position 236. N-terminal of GSDMB may lead to pyroptosis. Relevent studies suggest that N-terminal of GSDMB cannot form pores, and that the cell death associated with GSDMB is caused by Caspase-4. The combination of GSDMB and the CARD domain of Caspase-4 changes the conformation of Caspase-4, which in turn promotes the lysis of GSDMD, resulting in the occurrence of pyroptosis.

GSDMB, which is known as gasdermin-like protein, is composed of 411 amino acids and may cover genes that influence diseases associated with aberrant immune responses (Refs 39, 40). Human GSDMB has six splicing variants, each of which encodes a protein with a molecular weight ranging from 35 to 50 kDa that has different expression profiles and subcellular localization patterns in different cell types. Compared with other gasdermins, these isoforms are weaker and more unstable in terms of their functions and structures (Refs 41, 42). A recent study found that GSDMB can induce pyroptosis-like characteristics, but the mechanism whereby GSDMB activity leads to pyroptosis and participates in the regulation of inflammation needs to be further explored (Ref. 43). Chao *et al* (Ref. 41). found that GSDMB is not a substrate for inflammatory caspases 1 and 4/5/11 because of its lack of a specific interdomain linker region site. In addition, based on the phospholipid-binding activities of GSDMB and the cleavage profile of caspases, they confirmed that GSDMB proteins are substrates of the executioner caspases-3, -6 and -7, which activate apoptosis, not inflammatory caspases. Panganiban *et al* (Ref. 44) found that the expression of GSDMB alone does not stimulate pyroptosis. Further experiments indicated that GSDMB is cleaved by caspase-1 at site 236. One of the cleaved forms is the N-terminus of the GSDMB protein, which induces pyroptosis. Chen *et al* (Ref. 45) reported that the N-terminus of GSDMB does not cause pyroptosis by itself. The binding of full-length GSDMB to the CARD domain of caspase-4 may lead to the oligomerization of caspase-4, which in turn causes conformational changes in caspase-4, thus increasing its enzymatic activity and promoting the cleavage of GSDMD. They also proposed that in non-canonical pyroptosis, the effect of GSDMB on caspase-4 can be halted through a negative feedback mechanism, which may be an essential protective function during immune overreaction to infectious pathogens. Therefore, they believe that the N-terminus of GSDMB does not cause the formation of pores, and that GSDMB causes cell death by enhancing the enzymatic activity of caspase-4. The mechanism whereby GSDMB causes pyroptosis remains, however, to be elucidated. Different studies have reached different conclusions regarding the role of caspases in the cleavage of GSDMB in pyroptosis. Different cell lines and conditions may affect the cleavage site of caspases. However, only the cleavage of GSDMB by a specific caspase at a particular site can produce a pore-forming N-terminus, which may be the reason for the different conclusions. The specific process is shown in Figure 2.

**Fig. 3.**
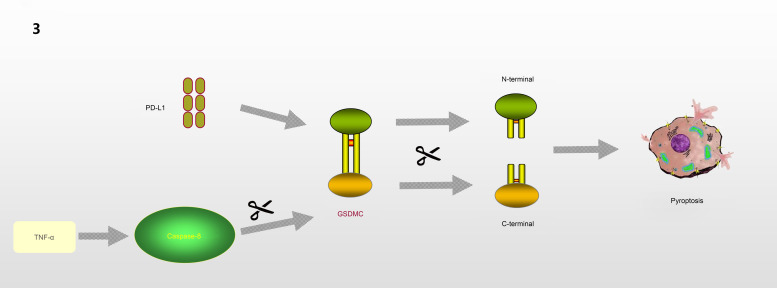
GSDMC is involved in the regulation of pyroptosis: In the presence of PD-L1, TNF-*α* induces Caspase-8 to cleave GSDMC at the site of 362LELD365 to generate N-terminal and C-terminal, which further leads to pyroptosis.

GSDMC, which is expressed in the epithelial cells of many tissues, is involved in controlling various cellular processes, including cell growth and death (Refs 37, 46–50). As illustrated in Figure 3, Hou *et al* (Ref. 51) demonstrated that the N-terminal domain of GSDMC can induce pyroptosis, and 362LELD365 is the site where caspase-8 cleaves GSDMC. Due to the presence of GSDMC and nuclear programmed death ligand 1 (nPD-L1), TNF-*α* activates caspase-8 and switches from apoptosis to pyroptosis, leading to tumour necrosis in hypoxic areas. Current research on GSDMC mainly focuses on lumbar spinal stenosis, melanoma, breast cancer, lumbar disc herniation, lung adenocarcinoma, chronic back pain and ultraviolet radiation (Refs 47–54).

**Fig. 4.**
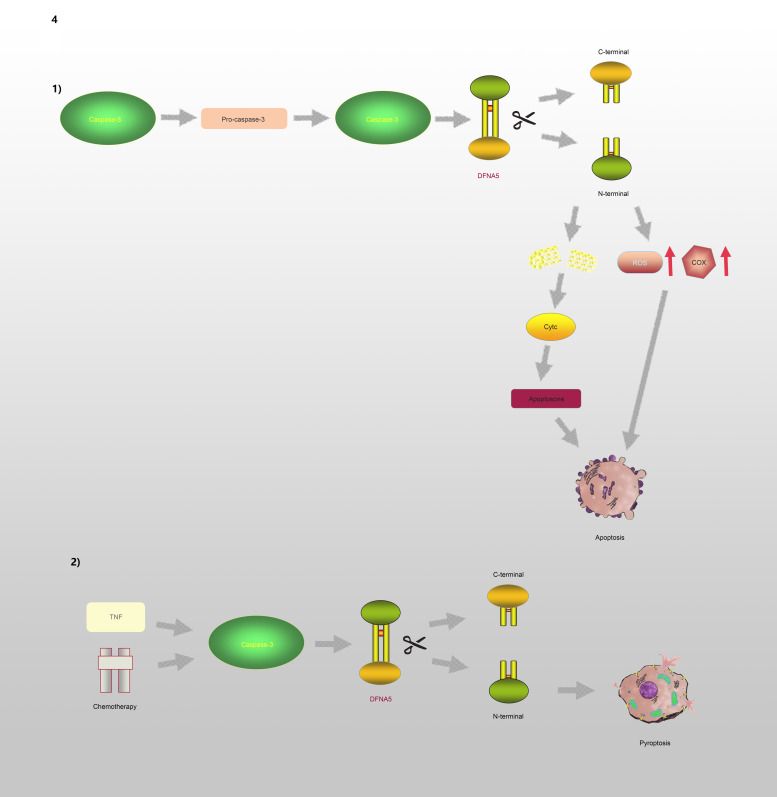
DFNA5 is involved in the regulation of apoptosis and pyroptosis: (1) Caspase-8 and Caspase-3 promote the cleavage of DFNA5 into N-terminal and C-terminal, leading to increasing levels of ROS and COX, mitochondrial damages, further causing generation of Cyct and apoptosome, and ultimately leading to apoptosis. (2) TNF or chemotherapeutic drugs induce Caspase-3 to cleave DFNA5 to generate N-terminal and C-terminal, resulting in the pore formation of cell membrane and pyroptosis.

GSDME, which is also known as DFNA5, has an apoptosis-inducing activity that depends on the domain structure (Refs 32, 55). A relevant study has illustrated that mutations in *DFNA5* significantly enhance ROS production and upregulate several cytochrome c oxidase genes involved in OS (Ref. 56). Moreover, endoplasmic reticulum stress, mitochondrial damage and the MAPK pathway are involved in DFNA5-induced cell death (Refs 57, 58). Further studies have revealed that the DFNA5-NT domain is associated with apoptosis and pyroptosis, which is shown in Figure 4. Caspase-3 is involved in cell apoptosis; however, Wang *et al* (Ref. 29) found that either TNF-*α* or chemotherapeutic drugs facilitate the cleavage of DFNA5, leading to the conversion of cell apoptosis to pyroptosis. DFNA5 can be specifically cleaved at its junction by caspase-3 to generate a DFNA5-NT fragment that penetrates the membrane and induces pyroptosis, which suggests that excessive apoptosis with caspase-3 activity can further lead to pyroptosis. Rogers *et al* (Ref. 30) also confirmed that DFNA5 mutation causes the mitochondria to release cytochrome C, activate caspase-3, and form pores in the plasma membrane. This means that regardless of whether caspase-1 is absent or non-functional, cells will continue undergoing pyroptosis even without GSDMD activation (Ref. 59).

**Fig. 5.**
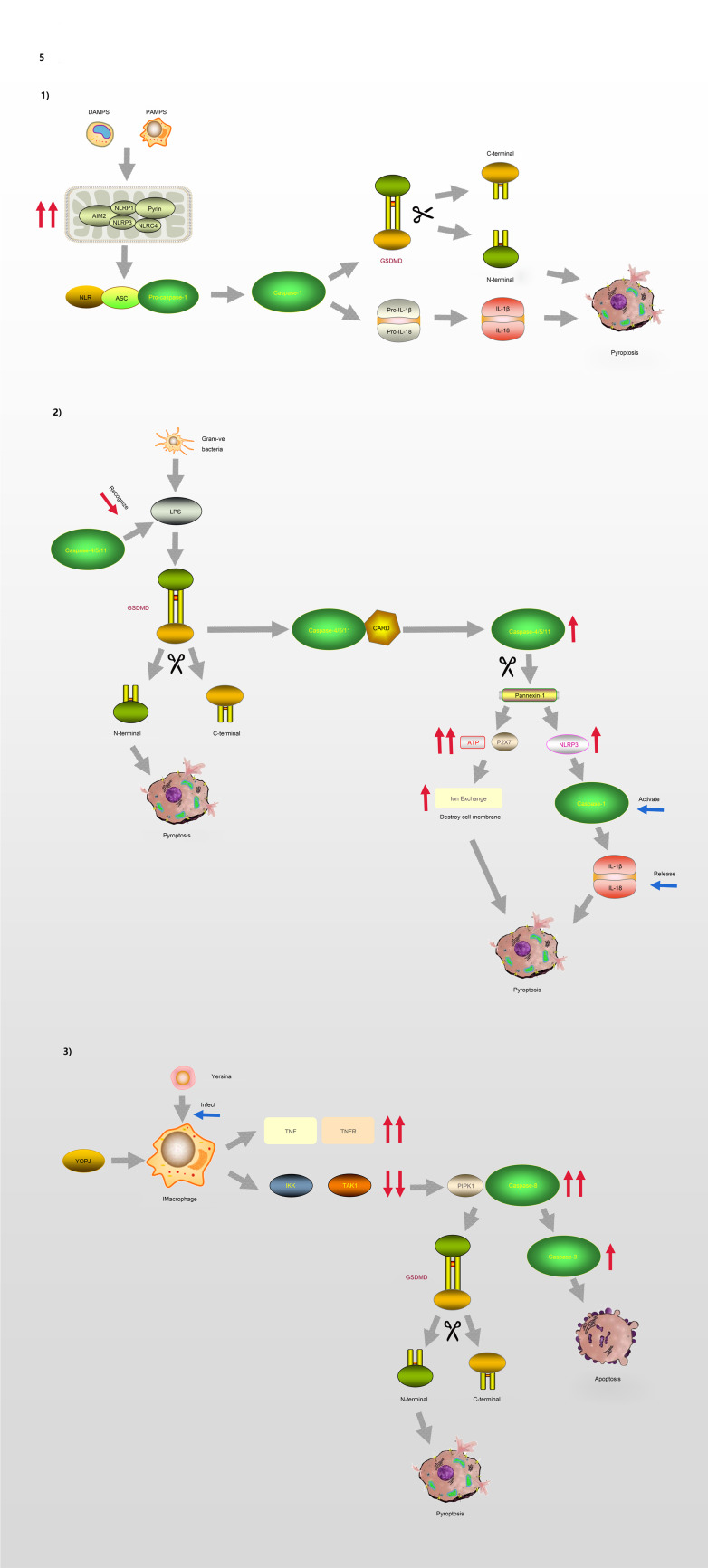
Pyroptosis and GSDMD. (1) Canonical pathway: The formation of classic inflammasome containing Caspase-1 on the one hand accelerates the release of IL-18 and IL-1*β*; on the other hand, it also directly lyses GSDMD, leading to cell membrane pore formation and pyroptosis. (2) Non-canonical pathway: Caspase-4/5/11 cleaves GSDMD and causes pyroptosis. The activation of Caspase-11 cleaves Pannexin-1, thereby destroying the integrity of the cell membrane. In addition, Pannexin-1 activates NLRP3 and participates in the formation of Caspase-1. (3) Model of macrophages infected by Yersina: After activated by Yersina, Caspase-8 cleaves GSDMD at the site of D276, which leads to pyroptosis or apoptosis by activating Caspase-3.

GSDMD is a key factor regulating pyroptosis (Refs 60–62). GSDMD is composed of a 242-amino acid N-terminal domain (GSDMD-NT) and a 199-amino acid C-terminal domain (GSDMD-CT) connected via a 43-amino acid linker. GSDMD-NT can bind to lipids, insert into the cellular membrane, and form pores (Ref. 61), GSDMD-NT is also known as the pore-forming domain. However, under non-stimulating conditions, its pore-forming activity is inhibited by GSDMD-CT; therefore, GSDMD-CT is also known as the repressing domain. In resting cells, the two aromatic amino acids Phe and Trp on the 1–2 loop of GSDMD-NT are bound to the same hydrophobic pocket located on the surface of its C-terminal domain and form autoinhibition structures. In such conditions, GSDMD is in a self-inhibiting state (Ref. 63). When pathogen-associated molecular pattern (PAMP) receptors, such as Toll-like receptors, recognise their ligands, inflammasomes are activated and GSDMD is cleaved by activated caspase-1. The N-terminal and C-terminal regions of GSDMD are dissociated, and the self-inhibiting structure disappears. Consequently, the N-terminus of GSDMD binds to membrane lipids and forms micropores with a diameter of 10–14 nm, resulting in cell rupture and the occurrence of an inflammatory cascade (Ref. 64). GSDMA, GSDMC and GSDMD are all capable of suppressing tumours, whereas GSDMB is considered an oncogene associated with immune diseases, such as childhood-onset asthma (Refs 65, 66). The molecular mechanism of GSDMD regulating the process of pyroptosis is illustrated in Figure 5.

### Process and signal transduction of pyroptosis

Currently, two pathways are known to be involved in the regulation of pyroptosis, including the conventional pathway mediated by caspase-1 and a non-canonical pathway regulated by caspase-4/5 (human) and caspase-11 (mouse), which senses intracellular bacterial lipopolysaccharide (LPS) (Refs 67, 68). In response to different types of stimulation, cells initiate pyroptosis through different pathways.

The process of pyroptosis begins with the assembly and integration of inflammasomes. The canonical pathway, which relies on caspase-1, is initiated by PAMP recognition and the activation of inflammasome sensors such as NLRP1b, NLRP3, NLRC4, AIM2 and pyrin (Ref. 69). Thus, inflammasome complexes are generated in immune cells, including macrophages and dendritic cells, under the stimulation of various signals. The complexes are composed of NOD-like receptor proteins (NLRPs) and a framework protein – apoptosis-associated speck-like protein containing a CARD (ASC), precursory caspase-1 (pro-caspase-1) and other related proteins. The framework protein ASC acts as a linker protein that can tightly connect NLRs and pro-caspase-1 via its N-terminal PYD and C-terminal CARD domains, respectively. Subsequently, pro-caspase-1 is activated and cleaved into two subunits, P20 and P10, forming the classic inflammasome containing caspase-1 (Refs 70, 71). On the one hand, upon meeting the inflammasomes, pro-caspase-1 self-cleaves into caspase-1, and because of its catalytic activity, pro-interleukin-1*β* (Pro-IL-1*β*) and pro-interleukin-18 (Pro-IL-18) are cleaved, resulting in the inflammatory factors interleukin-1*β* (IL-1*β*) and interleukin-18 (IL-18). On the other hand, activated caspase-1 can directly cleave GSDMD, causing the release of GSDMD-NT and consequently, leading to cell membrane pore formation and pyroptosis.

In the non-canonical signalling pathway, caspases-4 and -5 (human) or caspase-11 (mouse) can directly recognise LPS in the cytoplasm and cleave the GSDMD protein, resulting in pyroptosis (Ref. 72). After LPS-induced activation, caspase-11 cleaves pannexin-1, which controls the entry and exit of small molecules, leading to the release of ATP and the opening of the membrane channel P2X7, thereby mediating intracellular potassium efflux and the activation of the NLRP3 inflammasome (Refs 73, 74). In addition, pannesin-1 stimulates NLRP3 in apoptotic cells, promoting the release of IL-1*β* and the activation of caspase-1 (Refs 75, 76).

Moreover, caspase-8 is involved in the regulation of some infection-related immune pathways (Refs 77–80). A macrophage model of *Yersinia* infection demonstrated that the induction of pyroptosis by caspase-8 occurs via the hydrolysis and activation of gasdermin (Refs 81, 82). Orning *et al* (Ref. 81) confirmed that caspase-8 cleaves GSDMD at site D276, and suggested that after *Yersinia* activates caspase-8, GSDMD may not be the only downstream effector of caspase-8. These findings indicate that there is no strict boundary between the regulation of caspases and their downstream molecules, and that cross-pathway regulation exists in the face of different types of stimuli.

## GSDMD governed the pyroptosis involving in the development of DN

DN is an abnormality in the structure and function of the kidneys caused by diabetic chronic microvascular disease, which eventually leads to renal failure and ESRD. High glucose (HG) induces mitochondrial dysfunction, such as mitochondrial dynamics disorder, abnormal biosynthesis and DNA mutations. Various pathophysiological changes act on kidney cells, causing a series of inflammatory reactions. Intrinsic renal cells mainly include TECs and podocytes. The relationships between pyroptosis and DN in these two types of renal cells are described below.

### Tubular epithelial cell pyroptosis in DN

To maintain homeostasis, TECs are responsible for reabsorption of the kidneys, transferring some or all the water and several solutes from the tubules to the blood, retaining useful substances, and effectively removing harmful and excess substances. Under HG conditions, TECs are more susceptible to metabolic disorder, inflammation and haemodynamic changes, leading to the release of ROS and a variety of inflammatory factors, which results in renal interstitial inflammation and fibrosis. Relevant studies have reported that the pyroptosis of TECs occurs in the process of acute kidney injury and renal function damage caused by a contrast agent (Ref. 83), which suggests that pyroptosis participates in the occurrence and development of renal tubular damage in kidney disease.

Long non-coding RNAs (lncRNAs) are more than 200 nucleotides in length and only encode a small number of functional short peptides found in eukaryotes. They are important regulatory factors in the development of various diseases, and have been shown to be related to pyroptosis (Refs 84–86). MicroRNAs (miRNAs) are small endogenous non-coding RNAs with a length of approximately 22 nt, which are involved in cell proliferation and differentiation, programmed death, OS and the regulation of inflammation (Ref. 87). They are also recognised as important molecular regulators (Refs 88, 89). LncRNA combine with miRNA through the 3′-UTR region to form a ceRNA structure and participate in the regulation of related pathways. Previous studies have confirmed that lncRNA GAS5 expression is elevated in the serum of DM patients (Ref. 90). Xie *et al*. investigated the expression and relation of GAS5 and miR-452-5p in HG-induced HK-2 cells, and their effect on inflammation, OS and the pyroptosis of these cells (Ref. 91). They found that after HG treatment, GAS5 expression is reduced in HK-2 cells, while that of miR-452-5p is increased, and that GAS5 may directly target miR-452-5p. In addition, GAS5 overexpression was found to inhibit HG-induced inflammation, OS and pyroptosis through miR-452-5p interference. In this process, GSDMD-NT expression was shown to be upregulated in HG-induced HK-2 cells, which promoted pyroptosis. Moreover, the expression of lncRNA KCNQ1OT1 is increased in patients with diabetic cardiomyopathy, and its inhibition reduces the damage to cardiomyocytes (Ref. 92). On the other hand, it was found that miR-506-3p participates in the regulation of OS (Ref. 93). Zhu *et al* (Ref. 94) found that the expression of KCNQ1OT1 is increased in HG-induced HK-2 cells and in the plasma of DN patients. In addition, they confirmed that KCNQ1OT1 directly targets miR-506-3p using a luciferase assay. They showed that the expression of miR-506-3p is downregulated in HG-treated HK-2 cells and in the plasma of patients with DN, and that KCNQ1OT1 interference could reduce the levels of caspase-1, NLRP3 and GSDMD-NT by upregulating the expression of miR-506-3p, thereby alleviating inflammation, OS and the pyroptosis of HG-induced HK-2 cells. Therefore, KCNQ1OT1 may be a novel target for the treatment of DN. Metastasis-associated lung adenocarcinoma transcript 1 (MALAT1) is a lncRNA that is widely expressed in mammalian kidney tissues and increased in cancer cells (Ref. 95), and it is related to DM-related pyroptosis. Li *et al* (Ref. 96) found that MALAT1 expression is significantly increased, while that of miR-23c is decreased, in STZ-induced diabetic rats and HG-induced HK-2 cells. Downregulating the expression of MALAT1 or upregulating that of miR-23c can inhibit pyroptosis in HK-2 cells, and decrease the levels of NLRP3, caspase-1 and the inflammatory factor IL-1*β*, suggesting that the inhibition of MALAT1 can suppress HG-induced pyroptosis.

In addition to ceRNA, relative research has begun on the regulatory pathway mediated by Toll-like receptor 4 (TLR4). TLR4, a member of the TLR family, plays an important role in activating immune responses. It typically activates the nuclear factor-*κ*B (NF-*κ*B) pathway through MyD88, leading to the release of ROS – which causes OS – and cytokines. TLR4 induces the activation of caspase-1 and the expression of caspase-11, resulting in pyroptosis and the release of pro-inflammatory factors (Ref. 97). Wang *et al* (Ref. 98) examined TLR4 expression in DN patients and renal tubular cells, and found that increased TLR4 expression was related to renal tubular damage. They found that HG induces increased TLR4 expression, GSDMD cleavage and IL-1*β* release in vivo and in vitro, and that these effects can be reversed with TLR4 and NF-*κ*B inhibitors. These results indicate that the TLR4/NF-*κ*B signalling pathway is involved in regulating the pyroptosis of TECs in DN. In addition, Pang *et al* (Ref. 99) confirmed that nuclear factor erythroid-2 related factor 2 (Nrf2)/NLRP3 signalling is involved in the regulation of caspase-1-GSDMD-mediated pyrolysis in HK-2 cells. In addition, Nrf2, a protective factor in DN, attenuates ROS and regulates the redox balance under OS (Refs 100–102). It has been considered as a potential therapeutic target to prevent and reverse the progression of DN (Ref. 103).

Besides the above mechanisms, thioredoxin-interacting protein (TXNIP) regulating the level of ROS to mediate the process of pyroptosis is also an interesting direction. TXNIP is a negative regulator of thioredoxin (TRX). TXNIP–TRX interaction accelerates the generation of intracellular ROS and regulates redox reactions, which are closely related to the OS of the glomeruli (Refs 104, 105) and renal tubules (Ref. 106), and these processes are involved in the progression of DN (Ref. 107). Ke *et al* (Ref. 108) found that rat renal tubular epithelial NRK-52E cells are activated by the HG-induced TXNIP/NLRP3 axis, thereby inducing inflammation and pyroptosis. After inhibiting endoplasmic reticulum stress-related factors such as inositol-requiring enzyme 1 (IRE1), the expression of miR-200a increased and the expression of TXNIP decreased. Thus, IRE1 may mediate the pyroptosis and renal damage caused by the TXINP/NLRP3 pathway through the degradation of miR-200a. TXNIP also regulates the level of pyroptosis in DN by co-acting with ceRNA. Song *et al* (Ref. 109) confirmed that elevated levels of lncRNA ANRIL could induce vascular cell apoptosis, aggravate cell inflammation and lead to endothelial cell dysfunction in atherosclerosis. ANRIL and TXINP expression increases, and that of miR-497 decreases, after the induction of HG (Ref. 110). MiR-497, which binds to ANRIL and directly targets TXINP, was found to inhibit the expression of ANRIL, thereby suppressing the activation of TXNIP/NLRP3/caspase-1 and the release of IL-1*β* and IL-18. By restraining miR-497 or overexpressing TXNIP, the effects that ANRIL knockdown and inhibition of the pyroptosis were reversed (Ref. 110).

### Podocyte pyroptosis in DN

Podocytes – terminally differentiated cells located in the outer layer of glomerular capillaries – are composed of a cytoskeleton structure, joint connections and branched foot processes surrounding the glomerular capillaries. Once damaged, they cannot be regenerated. The basal, basolateral and parietal areas together constitute the foot process that adheres to the glomerular filtration barrier through the podoplanin protein. The reticular structure formed by the slit diaphragm between the foot process and the podocytes is involved in various signal transduction pathways in podocytes, which are essential for maintaining the structure of the glomerulus and the filtration function. Abnormal glomerular filtration and damage to podocytes are the core reasons for the development of proteinuria and glomerular sclerosis in DN (Ref. 111). Cheng *et al* (Ref. 112) found that HG intervention promoted caspase-11 and caspase-4 expression, and the decomposition of GSDMD. The knockout of caspase-11 or GSDMD could significantly improve the deterioration of renal function and the morphological changes of glomeruli and podocytes and alleviate the surging of inflammatory factors.

Mammalian target of rapamycin (mTOR), which is a highly conserved serine/threonine kinase that has been shown to regulate cell growth and proliferation in various in vivo and in vitro models, can combine with NLRP3 to regulate the level of inflammation (Refs 113–115). Studies have confirmed that the expression of NF-*κ*B is highly correlated with the mTOR signalling pathway (Ref. 116), the inhibition of which could protect podocytes and reverse DN by blocking the transdermal differentiation of glomerular mesangium (Ref. 117). Wang *et al* (Ref. 118) treated podocytes with an mTOR activator/inhibitor and an NF-*κ*B inhibitor, confirming that the mTOR/NLRP3/IL-1*β* axis is able to suppress podocyte damage in DN.

In addition to the mTOR/NLRP3 signalling pathway, mitochondrial function can also affect the level of inflammation and pyroptosis. SIRT1, a member of the mammalian silent information regulator (SIRT) protein family, is an important deacetylase in the mitochondria and participates in a variety of metabolic processes. Mitochondria can regulate the activation of the inflammasomes involved in pyroptosis (Refs 119, 120). Adenosine 5′-monophosphate (AMP)-activated protein kinase (AMPK) is involved in the regulation of energy metabolism. Activation of the AMPK/SIRT1 pathway can play a protective role in various inflammation-related diseases by inhibiting OS and apoptosis (Refs 121–123). AMPK has been reported to inhibit the expression of NF-*κ*B by increasing SIRT1 levels, thereby reducing inflammation (Ref. 124). Relevant studies have confirmed that AMPK, p-AMPK and SIRT1 levels are significantly reduced in HG-induced podocytes, and that the occurrence of DN may be related to the APMK/SIRT1/NF-*κ*B pathway (Refs 125, 126).

TNF-*α*-induced protein 3, also known as A20, is a protein encoded by the gene *TNFAIP3* that participates in the regulation of inflammatory signals by inhibiting NF-*κ*B, thereby exerting anti-inflammatory effects and protecting tissue cells (Ref. 127), and suppressing the activation of NLRP3 (Ref. 128). Many studies have shown that miR-21-5p promotes the development of inflammation (Refs 129, 130). In addition, miRNA transferred through extracellular vesicles can regulate mRNA levels after entering the cell, thereby influencing a variety of diseases (Refs 131, 132). Ding *et al* (Ref. 133) proved that miR-21-5p in macrophage-derived extracellular vesicles can regulate podocyte pyroptosis through A20.

The sponge structure formed by lncRNA and miRNA can also be involved in the regulation of podocyte pyroptosis. The lncRNA NEAT1 plays a vital role in the occurrence and development of DN; lncRNA NEAT1 expression is upregulated in both in vivo and in vitro models of DN (Ref. 134), and promotes epithelial-to-mesenchymal transition and kidney fibrosis in DN (Ref. 135). A meta-analysis of miRNA expression profiles in DN showed that at least two studies revealed the downregulation of miR-34c (Ref. 136). Recently, Liu *et al* (Ref. 137) found that miR-34c inhibits cell death in HG-induced podocytes. Zhan *et al* (Ref. 138) found that NEAT1 promotes podocyte pyroptosis by regulating miR-34c, whose target was shown to be NLRP3, thus regulating the expression of NLRP3, caspase-1 and IL-1*β*. These findings highlight the role of the NLRP3/caspase-1/IL-1*β* axis in DN. Zuo *et al* (Ref. 139) found that the level of MALAT1 and miR-200c increased, while the level of NRF2 decreased in the mouse podocytes treated with HG. MALAT1, as an upstream factor, affected the expression of miR-200c and NRF2. In addition, the level of OS also changed respectively by interfering with the expression of the three above. They confirmed that MALAT1/miR-200c/NRF2 axis is relative to regulating the pyroptosis and OS of podocyte treated with HG.

## Current clinical drugs and potential treatments for DN

The present treatment principles for DN are mainly to control blood sugar and blood pressure, protect kidney function, increase high-quality protein intake, reduce cardiovascular and cerebrovascular and peripheral vascular complications, etc. When it progresses to chronic renal failure, dialysis replacement therapy is needed, whose purpose is to delay its progress. At present, domestic and foreign studies on the clinical hypoglycaemic drugs to reverse the process of DN by inhibiting renal pyroptosis have not yet been fully established, which indicates that the pathogenesis of DN and the exploration of the drug mechanism are incomplete.

At the moment, it has been confirmed that dipeptidyl-peptidase-4 inhibitors saxagliptin inhibits the activation of NLRP3 inflammasomes, downregulates the expression of IL-18 and IL-1*β*, and avoids inflammation and renal fibrosis (Refs 140, 141). Although it was found that saxagliptin downregulates the level of pyroptotic factors in the process of inhibiting inflammation, whether it protects renal function by inhibiting pyroptosis still needs to be further explored.

Although the mechanism of clinical drugs in inhibiting pyroptosis has not yet been elucidated, potential therapeutic methods regarding their important role in controlling the level of pyroptosis have emerged. Necrosulfonamide (NSA), a pyroptosis inhibitor, has been proved to reduce the level of inflammation through various signalling pathways to inhibit the level of pyroptosis in many diseases such as non-small-cell lung cancer, gastric cancer, pulmonary fibrosis and coronary artery disease (Refs 142–145). It has great prospects, but whether it inhibits the level of renal pyroptosis and delays the deterioration of renal function has not yet been studied. Relative studies might be established to explore whether it plays a reversal role in renal pyroptosis caused by DN. It may become a new hope for the treatment of DN.

## Conclusion and outlook

Pyroptosis is one type of cell death that is associated with caspase-1 and accompanied by the releasing of inflammasomes. Under the action of different external stimuli, the cell membrane is punctured via the GSDMD protein through different channels, which leads to cell rupture, necrosis and the flowing out of cell contents. GSDMD-mediated pyroptosis is related to the occurrence and development of DN. This review summarises the relationships between the signalling pathways induced by HG in TECs and renal podocytes, and the process of GSDMD-mediated pyroptosis. However, the pathogenesis of DN is complicated, and many mechanisms are involved in pyroptosis. The specific regulatory network requires further investigation. Currently, the signalling pathways involved in the pyroptosis of other kidney cells, such as glomerular endothelial cells and glomerular mesangial cells, during DN are still unknown. With the gradual improvement of genetic research technology and the continuous expansion of our understanding of in vivo and in vitro functions, we may eventually gain a comprehensive understanding of the pathogenesis of DN caused by pyroptosis, which will result in a new breakthrough in its clinical treatment.
